# Hospital Statistics

**Published:** 1914-11-28

**Authors:** 


*To the Editor of* The Hospital.

### Hospital Statistics.

*To the Editor of* The Hospital.

Sir,—I recently had a discussion with a hospital official as to the correct way of ascertaining the following :—

(1) Average number of patients daily in hospital for any period.

(2) Average number of days each patient was under treatment for any period.

(3) Average cost per occupied bed for any period.

(4) Number of in-patients for a year or other period.

I should be greatly obliged if you would kindly state through the medium of the "Enquiries and Answers" column of your most valued Journal the true way in which to obtain the above-mentioned.—Yours faithfully,

Statistics.

November 11, 1914.

[All the above averages depend upon the daily counts—*i.e.*, the number of days collectively spent in hospital by in-patients. In No. 1 the answer is arrived at by dividing the number of hospital days (daily counts) by the number of days the hospital was open during the year or other period. This will give the daily average number of beds occupied, which is the same as question (1). (2) is found by dividing the number of hospital days (daily counts) by the number of in-patients treated to a conclusion; (3) by dividing the total cost of in-patients, excluding rent, rates, and taxes, by the daily average number of beds occupied; (4) is the number of in-patients in hospital at the beginning of the year or other period *plus* those admitted during the period in question, *minus* those remaining in hospital at the end of the year or other period.—Ed. The Hospital.]

